# Racial differences in the burden of coronary artery calcium and carotid intima media thickness between Blacks and Whites

**DOI:** 10.1007/s12471-014-0610-4

**Published:** 2014-10-24

**Authors:** S. Erqou, K. E. Kip, S. R. Mulukutla, A. N. Aiyer, S. E. Reis

**Affiliations:** 1 Department of Medicine, Weill Cornell Medical College, 525 E 68th Street, Box 130, New York, NY 10065 USA; 2 College of Nursing, University of South Florida, 4202 E. Fowler Avenue, Tampa, FL 33620 USA; 3 Heart and Vascular Institute, University of Pittsburgh Medical Centre – Presbyterian Hospital, 200 Lothrop Street, PUH, Room A-341, Pittsburgh, PA 15213 USA; 4 Cardiovascular Institute at University Centre, 120 Lytton Avenue, Suite 302, Pittsburgh, PA 15213 USA; 5 Department of Medicine, University of Pittsburgh, Scaife 401, 3550 Terrace Street, Pittsburgh, PA 15216 USA

**Keywords:** Carotid intima media thickness, Coronary artery calcium, Subclinical atherosclerosis, Racial-disparity, Risk factor, Observational study

## Abstract

**Background:**

Identification of racial differences in the burden and correlates of carotid intima media thickness (CIMT) and coronary artery calcium (CAC) may provide the basis for the development of race-specific cardiovascular disease (CVD) risk prediction algorithms.

**Methods:**

In the Heart Strategies Concentrating on Risk Evaluation (Heart SCORE) study, CIMT was measured by carotid ultrasonography in 792 individuals (35 % Black). CIMT >1 mm was considered significant. CAC was quantified by electron beam computed tomography in 776 individuals (46 % Black). CAC was considered significant if the Agatston score was >100. Cross-sectional associations between race, CIMT and CAC were assessed using logistic regression models.

**Results:**

Blacks had greater CIMT (mean difference 0.033 mm, 95 % CI 0.005–0.06 mm; *p* = 0.02) and 1.5-fold (95 % CI 1.0–2.3) higher odds of having significant CIMT than Whites. Blacks had less CAC than Whites (mean Agatston score difference 66, [11–122]; *p* = 0.02) and 50 % lower odds of a significant CAC score compared with Whites (0.5 [0.3–0.7]). These associations were virtually unchanged after adjustment for CVD risk factors. Of the novel CVD risk markers assessed, small-dense low-density lipoprotein was independently associated with increased odds of significant CIMT, with the association being similar among Blacks and Whites (odds ratio [95 % CI]: 1.7 [1.2–2.5] and 1.4 [1.0–1.8] per 1-SD higher level, respectively). Interleukin-6 was significantly associated with CAC among Blacks (1.4 [1.0–2.0]).

**Conclusion:**

Black race is independently associated with greater CIMT but less CAC than White race. CVD risk stratification strategies that incorporate these measures of subclinical atherosclerosis should consider race-specific algorithms.

**Electronic supplementary material:**

The online version of this article (doi:10.1007/s12471-014-0610-4) contains supplementary material, which is available to authorized users.

## Introduction

Blacks have a >2-fold increased risk of stroke, a similar risk of coronary artery disease (CAD) morbidity, and a higher risk CAD mortality compared with Whites [1–4]. These cardiovascular disease (CVD) disparities are not fully explained by differences in traditional CVD risk factors, even though Blacks have more prevalent hypertension and diabetes [5, 6]. Therefore, evaluation of race-related differences in measures of subclinical atherosclerosis, which serve as surrogates for cardiovascular disease (CVD), and the association between these measures and both traditional and novel CVD risk factors may provide a better understanding of racial disparities in CVD and support the development of race-specific risk stratification algorithms [6, 7].

Carotid intima media thickness (CIMT) and coronary artery calcium (CAC) score are two well-established measures of subclinical atherosclerosis that are used to stratify future risk of CVD events [6, 8–16]. Previous studies have reported inter-ethnic differences in the burden of CIMT and CAC. For instance, Blacks have a higher CIMT burden than Whites [6, 17, 18], although it is unclear whether this finding correlates with a greater burden of carotid plaque [17]. In contrast, we and others have reported a lower CAC burden in Blacks [9, 10, 19–21], although this finding is controversial [22, 23]. As in the case of stroke and CAD, observed inter-racial differences in CIMT and CAC burden are not fully explained by traditional CVD factors [6, 9, 10, 24]. Accordingly, we analysed data from the Heart Strategies Concentrating on Risk Evaluation (Heart SCORE) study [7, 9] to provide insight into race-specific CVD risk prediction. Specifically, we investigated inter-racial differences in CIMT and CAC burden, and their association with traditional CVD risk factors and novel risk markers.

## Methods

### Study population

Heart SCORE is an ongoing community-based prospective cohort study of racial disparities of CVD with approximately equal representation of Blacks and Whites. The methods of Heart SCORE have been described previously [7, 9]. Eligibility criteria included age 45 to 75 years at study entry, residence in the greater Pittsburgh metropolitan area, ability to undergo baseline and annual follow-up visits, and absence of known comorbidities expected to limit life expectancy to less than 5 years. The present report is based on cross-sectional analyses of 1,245 participants who had available information on either CIMT or CAC, including 327 individuals with information on both variables. The Institutional Review Board at the University of Pittsburgh approved the study protocol, and all study subjects provided written informed consent.

### Data collection

Detailed demographic and medical histories were collected at the baseline visit. Race was self-reported. Physical examination included measurement of vital signs and anthropometric measures of body fat distribution. Body mass index (BMI) was calculated as weight/height^2^ (kg/m^2^). Diabetes mellitus was defined as fasting glucose >126 mg/dl based on clinical guidelines at the time of study initiation or a history of previously diagnosed diabetes treated with diet, oral agents, and/or insulin.

Laboratory assessments of lipoprotein levels and particle sizes were performed on venous blood drawn in the fasting state. The amount of cholesterol contained in each lipoprotein particle subfraction was quantified by a commercial laboratory using the vertical auto profile technique (VAP, Atherotech, Birmingham, AL). This method was also used to quantify concentrations of proatherogenic, small-dense low-density lipoprotein (sdLDL). Fasting blood glucose was measured using the glucose oxidase method. Measurement of high-sensitivity C-reactive protein (hsCRP) was performed using an immunoturbidimetric assay on the Roche P Modular system (Roche Diagnostics - Indianapolis, IN), using reagents and calibrators from DiaSorin (Stillwater, MN). Concentrations of serum interleukin-6 (IL-6), soluble intercellular adhesion molecule 1 (sICAM-1), CD40 ligand (CD40L) and endostatin were measured using commercially available ELISA assay kits (R&D Systems, Minneapolis, MN). Measurements were performed using a standard curve provided with the ELISA kits; samples were read spectrophotometrically in a microtitre plate reader. To confirm reproducibility as reported by the kit manufacturer, a random subset of samples (10 %) was assayed in duplicate. The selection of the novel markers measured in this study was based on information from previous reports of associations between CVD and various pro-inflammatory cytokines, soluble cellular adhesion molecules and growth regulatory factors [25–28].

### Carotid artery imaging

Carotid artery imaging was carried out using a GE VIVID7 (General Electric Corp.) ultrasound imaging system and a 7 MHz linear array vascular ultrasound probe. The ultrasound beam was adjusted to obtain longitudinal scans of the carotid arteries to visualise two parallel echogenic lines corresponding to the blood-intima and media-adventitia interfaces on the posterior wall. End-diastolic images were recorded using electrocardiographic gating. Software on the GE VIVID 7 was used to calculate intima media thickness using automated edge detection to locate the lumen-intima and media-adventitia echo boundaries at subpixel resolution [29]. Media thickness averaged over 70–100 individual measurements taken along a 1-cm segment of the common carotid artery beginning 0.5 cm from the carotid bifurcation along the far wall of the distal common carotid artery. Significant CIMT was defined as maximal CIMT >1 mm in either the right or left carotid artery, as previously described by others [29].

### Coronary artery calcium measurement

Electron beam computed tomography image acquisition was obtained with Imatron C150 scanner (GE Imatron Inc, South San Francisco, CA) using 3-mm intervals to span the heart during a single inspiratory breath-hold. Calcium scores were calculated by the Agatston method using a densitometric program [30]. Scans were interpreted by an experienced reader blinded to subject identities. Significant CAC scores were defined as an Agatston score of >100, which corresponds to significant CAC burden, as previously described [9, 30].

### Statistical analysis

Baseline characteristics of Black and White participants were compared using chi-squared tests (for categorical variables) or Student’s *t* test (for continuous variables). Variables with skewed distributions (i.e., CRP and IL-6) were log-transformed to approximate normal distribution, before applying the *t*-test or fitting in regression models. The association of race with presence of significant CIMT or CAC was assessed using logistic regression models. Covariates used for statistical adjustment included conventional CVD risk factors (age, sex, smoking, BMI, systolic blood pressure, LDL-cholesterol, HDL-cholesterol and log-triglycerides), and novel risk markers (sdLDL, CRP, IL-6, sICAM-1, CD40L and endostatin). All analyses were performed using Stata software (Stata Corp., version 11, Texas, USA).

## Results

### Demographics

Data on CIMT and CAC were available on 792 (514 White, 278 Black) and 776 (414 White, 362 Black) participants, respectively. Of these, 327 participants underwent both CIMT and CAC measurement. Overall, the mean age of participants was 60 years with 42 % males and 11 % smokers. The characteristics of the participants subgrouped by race are shown in Tables 1a and b. As shown in these tables, White participants were older than the Black participants. Also, the proportion of males was higher among the White participants. Blacks had a significantly higher prevalence of diabetes, as well as higher mean BMI and systolic blood pressure, and higher CRP and IL-6 levels but lower levels of triglycerides and sdLDL, compared with Whites (these differences persisted in multivariable adjusted regression models including age, sex, smoking status and diabetes). Supplementary Tables 1a and 1b show the characteristics of the participants subgrouped by sex.

**Table 1:** a: Baseline characteristics of 792 participants with available information on carotid intima media thickness (CIMT) by race. b: Baseline characteristics of 776 participants with available information on coronary artery calcium (CAC) score by race

Variable	Whites		Blacks		*p*-value
	No of subjects	Mean (SD) or N (%)	No of subjects	Mean (SD) or N (%)	
a) Individuals with available data on CIMT					
Maximum CIMT (mm)	514	0.81 (0.19)	278	0.84 (0.18)	0.019
Age (years)	514	60 (7)	278	58 (7)	<0.001
Male	514	205 (40 %)	278	83 (30 %)	0.005
Current smoker	513	30 (6 %)	278	24 (9 %)	0.14
History of diabetes	510	22 (4 %)	277	31 (11 %)	<0.001
Systolic blood pressure	514	133 (18)	278	140 (20)	<0.001
Body mass index	506	28 (5)	274	32 (6)	<0.001
LDL cholesterol (mg/dl)	514	143 (34)	278	140 (40)	0.17
HDL cholesterol	514	55 (17)	278	58 (16)	0.08
Triglycerides (mg/dl)	514	134 (83)	278	105 (48)	<0.001
Fasting glucose (mg/dl)	511	96 (18)	277	99 (27)	0.10
sdLDL cholesterol (mg/dl)	514	48 (19)	278	43 (19)	<0.001
log-CRP (log-mg/dl)	487	0.13 (1.14)	257	0.49 (1.21)	<0.001
log-IL6 (log-mg/dl)	480	0.31 (0.77)	250	0.66 (0.68)	<0.001
CD40L (ng/ml)	359	2.75 (2.79)	184	1.55 (1.68)	<0.001
sICAM-1 (ng/ml)	429	232 (70)	234	197 (97)	<0.001
Endostatin (ng/ml)	379	125 (34)	199	123 (44)	0.50
b) Individuals with available data on CAC					
Total CAC score	414	190 (391)	362	124 (399)	0.01
Age (years)	414	61 (7)	360	60 (7)	0.001
Male	414	258 (62 %)	360	163 (45 %)	< 0.001
Current smokers	413	47 (11 %)	358	65 (18 %)	0.01
Diabetes history	411	49 (12 %)	359	97 (27 %)	<0.001
Systolic blood pressure	414	140 (20)	360	146 (19)	<0.001
Body mass index	410	30 (6)	359	32 (6)	<0.001
LDL cholesterol (mg/dl)	414	146 (36)	360	144 (41)	0.47
HDL cholesterol	414	48 (14)	360	52 (16)	<0.001
Triglycerides (mg/dl)	414	159 (106)	360	121 (71)	<0.001
Fasting glucose (mg/dl)	411	101 (21)	359	108 (40)	<0.001
sdLDL cholesterol (mg/dl)	418	53 (20)	362	48 (20)	<0.001
Log-CRP (log-mg/dl)	389	0.22 (1.10)	340	0.70 (1.21)	<0.001
Log-IL6 (log-mg/dl)	384	0.43 (0.73)	334	0.78 (0.66)	<0.001
CD40L (ng/ml)	137	2.47 (2.74)	90	1.70 (1.61)	0.017
sICAM-1 (ng/ml)	353	244 (85)	311	197 (104)	<0.001
Endostatin (ng/ml)	149	124 (34)	96	127 (44)	0.59

### Race and CIMT

Blacks had higher CIMT than Whites (mean difference 0.033 mm, 95 % CI 0.005–0.06 mm; p =0.02) and a 1.5-fold (95 % CI 1.0–2.3) greater odds of having significant CIMT. These higher odds were virtually unchanged after adjustment for conventional CVD risk factors (odds ratio [95 % CI]: 1.6 [1.0–2.6]; Table 2). The association of traditional CVD risk factors with CIMT is shown in Supplementary Table 2; in particular age, sex and smoking were strongly associated with risk of significant CIMT. Of the novel factors assessed, sdLDL was associated with higher odds of having significant CIMT after adjustment for CVD risk factors, with the association being relatively similar among Blacks and Whites (1.7 [1.2–2.5] and 1.4 [1.0–1.8] per 1-SD higher sdLDL level, respectively) (Table 3).

**Table 2:** The association of race with adjusted odds of significant carotid intima media thickness (>1 mm) and coronary artery calcification (Agatston Score >100)

Model and covariates	Significant carotid intima media thickness (>1 mm)			Coronary artery calcification (Agatston Score >100)		
	No. available	OR (95 % CI) for Black vs. White	*χ* ^2^	No. available	OR (95 % CI) for Black vs. White	*χ* ^2^
Crude	792	1.51(1.01,2.26)	4.1	776	0.47(0.34,0.65)	21.7
Adjusted for age and sex	792	1.91(1.26,2.92)	9.1	776	0.59(0.42,0.83)	9.0
Above plus adjusted for SBP, smoking, diabetes, and BMI	776	1.59(1.02,2.47)	4.2	767	0.49(0.34,0.70)	15.3
Above plus adjusted for total cholesterol, HDL-c, triglycerides and fasting glucose level	772	1.62(1.02,2.57)	4.2	763	0.50(0.35,0.73)	13.0

**Table 3:** The association of novel risk markers with adjusted odds* of significant carotid intima media thickness (>1 mm) and coronary calcification (Agatston Score >100), overall and by race

Marker		Significant carotid intima media thickness (>1 mm)			Significant coronary artery calcification (Agatston Score >100)		
		N available	OR (95 % CI) per 1-SD higher level	*χ* ^2^	N available	OR (95 % CI) per 1-SD higher level	*χ* ^2^
a) Overall association							
sdLDL		776	1.43(1.14,1.79)	9.7	767	0.92(0.77,1.10)	0.8
CD40L		531	0.96(0.71,1.29)	0.1	223	1.06(0.74,1.52)	0.1
Log-CRP		725	1.00(0.77,1.30)	0.0	716	1.05(0.84,1.30)	0.2
Log-IL-6		711	1.20(0.93,1.54)	2.0	704	1.28(1.04,1.58)	5.2
Endostatin		565	0.90(0.69,1.16)	0.7	240	1.04(0.75,1.44)	0.0
sICAM-1		648	0.84(0.63,1.12)	1.4	654	1.07(0.88,1.31)	0.5
Race	Variable	Significant carotid intima media thickness (>1 mm)			Significant coronary artery calcification (Agatston Score >100)		
		N available	OR (95 % CI) per 1-SD higher level	*χ* ^2^	N available	OR (95 % CI) per 1-SD higher level	*χ* ^2^
b) Association by race							
White	sdLDL	503	1.36(1.01,1.83)	4.2	410	0.98(0.78,1.24)	0.0
	CD40L	349	0.89(0.62,1.28)	0.4	133	1.16(0.77,1.74)	0.5
	Log-CRP	473	1.03(0.72,1.46)	0.0	380	1.03(0.77,1.39)	0.1
	Log-IL-6	466	1.21(0.88,1.66)	1.3	375	1.19(0.91,1.56)	1.6
	Endostatin	368	0.91(0.60,1.37)	0.2	144	0.93(0.59,1.45)	0.1
	sICAM-1	405	0.99(0.63,1.55)	0.0	346	1.06(0.79,1.41)	0.1
Black	sdLDL	273	1.73(1.19,2.50)	8.3	357	0.84(0.63,1.12)	1.4
	CD40L	182	1.16(0.65,2.08)	0.3	82	1.06(0.35,3.16)	0.0
	Log-CRP	252	0.95(0.63,1.43)	0.1	336	1.04(0.74,1.45)	0.0
	Log-IL-6	245	1.21(0.79,1.84)	0.8	329	1.40(0.98,1.98)	3.5
	Endostatin	197	0.84(0.58,1.20)	0.9	88	1.57(0.80,3.07)	1.7
	sICAM-1	230	0.75(0.51,1.11)	2.1	308	1.06(0.79,1.41)	0.2

*Adjustment for age, sex, race, systolic blood pressure, smoking status, diabetes, body mass index, total cholesterol, HDL-c, triglycerides and fasting glucose

Total cholesterol was not adjusted for when assessing the association of sdLDL

### Race and CAC

Blacks had lower CAC than Whites (mean Agatston score difference 66, 11–122; *p* = 0.02) and a 50 % lower odds of a significant CAC score (odds ratio [95 % CI]: 0.5 [0.3-0.7]). The association was unchanged after adjustment for CVD risk factors (0.5 [0.3–0.7]; Table 2). The association of traditional CVD risk factors with CAC is shown in Supplementary Table 2; in particular age and sex were strongly associated with risk of significant CAC. Of the novel factors, IL-6 was significantly associated with CAC (1.3[1.0, 1.6]). This association was evident among Blacks (1.4 [1.0–2.0]) and trended among Whites (1.2 [0.9–1.6]) (Table 3).

### Subsidiary analyses

Sensitivity analyses of 327 (205 White, 122 Black) participants with available information on both CIMT and CAC yielded similar results, although the association of race with CIMT was not statistically significant in the fully adjusted model, partly due to limited statistical power (Table 4). In this subset, individuals with significant CIMT (>1 mm) had higher risk of having significant CAC (odds ratio [95 % CI]: 2.2 [1.3–3.9]); however, this association was no longer statistically significant after adjustment for age, sex, race and conventional CVD risk factors (1.5 [0.8–2.8]) (data not presented in tables).

**Table 4:** The association of race with adjusted odds of significant carotid intima media thickness (>1 mm) and coronary artery calcification (Agatston Score >100) in a subset of 327 participants with available information on both measures of subclinical atherosclerosis

Model and covariates	Significant carotid intima media thickness (>1 mm)			Significant coronary artery calcification (Agatston Score >100)		
	*N* available	OR (95 % CI) for Black vs. White	*χ* ^2^	*N* available	OR (95 % CI) for Black vs. White	*χ* ^2^
Crude	327	1.03(0.59,1.80)	0.0	327	0.44(0.26,0.74)	9.6
Adjusted for age and sex	327	1.35(0.75,2.43)	1.0	327	0.50(0.29,0.88)	5.9
Above plus adjusted for SBP, smoking, diabetes, and BMI	323	1.24(0.67,2.30)	0.4	323	0.37(0.20,0.68)	10.2
Above plus adjusted for total cholesterol, HDL-c, triglycerides, and fasting glucose level	323	1.37(0.71,2.67)	0.9	323	0.35(0.18,0.65)	10.8

## Discussion

Our data from more than 1,200 participants in a study of racial disparities in CVD indicate that Black race is associated with higher CIMT but less CAC than White race. These associations were independent of traditional CVD risk factors. Of the novel CVD risk factors that were investigated, sdLDL was found to be significantly associated with CIMT but not with CAC. Interleukin-6 concentration was significantly associated with higher CAC in Blacks, and trended in the same direction in Whites.

Racial differences in the burden of subclinical atherosclerosis, which is a surrogate marker of CVD, have been previously reported [6, 10, 18–21]. For instance, Kanaya et al. compared US Asians in the MASALA study with Whites, African Americans, Latin Americans and Chinese Americans in the MESA study. They found that South Asians and Whites have similar CAC scores, but that their CAC scores were higher than those in the other ethnic groups, including African Americans [31]. Our findings of higher CAC burden in Whites than Blacks are consistent with their report. Moreover, our study complements previous studies on the topic because we concomitantly investigated CIMT and CAC in a racially diverse study population and determined their associations with both conventional and novel CVD risk factors. Our study confirms significant inter-racial differences in CIMT and CAC, and reports their correlations with traditional and novel CVD risk factors. The present report builds on our previous work that demonstrated a difference in CAC burden between Blacks and Whites [7, 9] in that we studied CIMT, in addition to CAC, and were able to assess their associations with race and several novel inflammatory markers.

In our study, higher levels of IL-6 were associated with a greater burden of CAC in Blacks, and showed a trend in Whites. Also, higher levels of sdLDL concentrations were significantly associated with greater burden of CIMT in both Blacks and Whites. Of note, this association appeared stronger in Blacks than Whites (adjusted odds ratio = 1.7 versus 1.4), but the difference was not statistically significant. Compared with large buoyant LDL particles, sdLDL particles have increased penetration into the arterial wall and increased susceptibility to oxidation. Hence sdLDL is believed to be more pro-atherogenic than larger LDL particles [32]. Similarly, inflammation is known to play an important role in the progression of atherosclerotic plaque [33, 34]. For example, recent genetic studies have shown IL-6 to be a likely causal factor in CVD [35]. In our study, we found that sdLDL and IL-6 were associated with CIMT and CAC, respectively. Given the higher burden of CIMT and lower burden of CAC in Blacks, one would expect higher levels of sdLDL and lower levels of IL-6 in Blacks compared with Whites. However, our findings demonstrate the opposite; Blacks had significantly lower levels of sdLDL and higher levels of IL-6. This is likely because CAC and CIMT are multifactorial surrogates of CVD that are determined by the interaction of several variables, besides sdLDL and IL-6 concentrations. In addition, the concentrations sdLDL and IL-6 in blood may influence other characteristics of CAC and CIMT, such as ‘plaque vulnerability’ (see below). We also found that sICAM-1 and CD40L were not associated with either CIMT or CAC, in keeping with previous studies showing lack of association of these novel risk markers with CVD [25, 33].

Coronary artery calcium burden has been shown to be a predictor of CVD risk [11, 15, 16]. Therefore, it is surprising that Blacks, who have higher rates of stroke and CVD mortality than Whites, have lower burdens of CAC than Whites as reported in previous epidemiological studies [10, 19–21]. Our study similarly found a substantially lower burden of CAC in Blacks compared with Whites. One proposed hypothesis for this paradoxical observation is that the level of CAC may not only be determined by traditional CVD risk factors, but also by certain aspects of calcium and vitamin D metabolism [36, 37]. For instance, Blacks as a group, have lower levels of vitamin D than Whites [38, 39]. Regardless of the mechanistic explanation for the lower calcium scores in Blacks, it is apparent that the use of the currently accepted cut-offs of CAC scores to predict CVD risk are more relevant to Whites than Blacks. On the other hand, the generally higher concentrations of inflammatory mediators (e.g., hsCRP, IL-6) observed in Black participants in our study, and the established role of inflammation in atherosclerotic plaque instability [40–42], suggests that the increased risk of CVD in Blacks may be related to higher plaque vulnerability of less calcified coronary lesions. Therefore, our data support previous suggestions that Agatston score cut-offs for risk categories are most clinically relevant if defined separately by race [43, 44]. Additional data from large prospective studies that measure CAC, CVD risk factors, and incident CVD outcomes concomitantly in both Blacks and Whites would be required to establish such cut-points. The determination of such ethnicity-specific cut-points may improve CVD risk stratification and preventive measures.

As with CAC, CIMT is a widely accepted indicator of CVD risk and has been shown to predict future risk of stroke and coronary events [8, 12–14]. In contrast to overall lower levels of CAC in Blacks compared with Whites, several studies, including ours, have demonstrated higher burden of CIMT in Blacks than Whites [6, 18]. These Black-White differences in CIMT have also been observed in children and adolescents, indicating that it is less likely to be explained by traditional CVD risk factors such as smoking [24]. A recent meta-analysis of eight observational studies showed that a 0.1 mm higher CIMT was associated with about a 15 % higher risk of CVD [8]. The mean racial difference in CIMT in our study was only 0.03, and the mean CIMT among Whites was greater than previously reported (0.81 vs. 0.75 mm) [17]. However, a substantial proportion of Blacks had CIMT >1 mm, compared with Whites, suggesting that the majority of the inter-racial disparity in CIMT may exist at the higher end of the spectrum. This finding supports the need for large prospective epidemiological studies of CVD outcomes to assess race-specific algorithms of CIMT as a predictor of CVD risk.

The strengths of the current study merit consideration. First, the study assessed CIMT and CAC in a general population composed of two races (Blacks and Whites), thereby allowing better characterisation of their relationships with race. Second, with >700 participants in each subset, the study was adequately powered to assess associations between race and subclinical atherosclerosis. Third, we had available data on a range of traditional and novel CVD risk factors thereby enabling detailed exploration of their role in the observed associations.

Our study also has a number of limitations. First, only a subset of our subjects had available data on both CIMT and CAC. Hence the separate analyses performed on CIMT and CAC were not directly comparable. However, sensitivity analyses of 327 participants with information on both CIMT and CAC yielded similar results. Second, the cross-sectional nature of our study limits the degree of inference that may be drawn. Third, due to lack of tabulation of ‘hard’ CVD outcomes, it is not possible to make specific recommendations of race-specific algorithms for characterising incident CVD risk using CIMT and CAC. Finally, we did not investigate certain aspects of inter-ethnic differences in cardiac variables, such as differences in cardiac morphology, which may influence CVD outcomes [45].

In conclusion, the current study provides evidence that Black race is associated with greater CIMT but less CAC than White race independent of conventional CVD risk factors. Small-dense LDL was associated with significant CIMT in both races, while IL-6 was associated with significant CAC burden in Blacks. The results suggest that additional studies are needed to develop race-specific algorithms for CVD risk stratification strategies that incorporate measures of subclinical atherosclerosis.

## Electronic supplementary material

Below is the link to the electronic supplementary material.

Supplementary table 1a (DOC 47 kb)

Supplementary table 1b (DOC 47 kb)

Supplementary table 2 (DOC 35 kb)
