# Supplementary material for: Racial differences in the burden of coronary artery calcium and carotid intima media thickness between Blacks and Whites
Source: Neth Heart J. 2014 Oct 24;23(1):44–51. doi: 10.1007/s12471-014-0610-4 (PMC4268220; doi:10.1007/s12471-014-0610-4)
Supplement: Supplementary file 1 — (DOC 47 kb) [file 12471_2014_610_MOESM1_ESM.doc]

Supplementary table 1a: Baseline characteristics of 792 participants with available information on carotid intima media thickness (CIMT) by Sex

| Variable | Male | | Female | | p-value |
| --- | --- | --- | --- | --- | --- |
|  | No of subjects | Mean (SD) or % | No of subjects | Mean (SD) or % |  |
| Max of IMT | 189 | 0.87 (0.19) | 504 | 0.79 (0.18) | <0.001 |
| Age (years) | 189 | 60 (7) | 504 | 60 (7) | 0.95 |
| Race – White | 189 | 135 (71%) | 504 | 309 (61%) | 0.005 |
| Race – Black | 189 | 54 (29%) | 504 | 195 (39%) |  |
| Current smoker | 189 | 15 (8%) | 503 | 33 (7%) | 0.70 |
| Diabetes | 189 | 11 (6%) | 500 | 32 (6%) | 0.62 |
| Systolic BP | 189 | 138 (18) | 504 | 135 (19) | 0.051 |
| Body mass index | 186 | 29 (4) | 498 | 30 (6) | 0.98 |
| LDL cholesterol (mg/dl) | 189 | 136 (33) | 504 | 146 (37) | <0.001 |
| HDL cholesterol | 189 | 46 (13) | 504 | 61 (16) | <0.001 |
| Triglycerides (mg/dl) | 189 | 132 (76) | 504 | 121 (72) | 0.16 |
| Fasting glucose | 189 | 101 (23) | 501 | 95 (21) | 0.002 |
| sdLDL (mg/dl) | 189 | 51 (18) | 504 | 44 (20) | <0.001 |
| Ln-hsCRP | 177 | 0.040 (1.005) | 473 | 0.38 (1.20) | <0.001 |
| Ln-IL6 | 174 | 0.36 (0.73) | 464 | 0.47 (0.76) | 0.05 |
| CD40L (ng/ml) | 118 | 2.04 (2.43) | 361 | 2.51 (2.56) | 0.026 |
| sICAM-1 (ng/ml) | 156 | 209 (73) | 421 | 221 (84) | 0.41 |
| Endostatin (ng/ml) | 128 | 121 (38) | 383 | 126 (38) | 0.13 |
