# Supplementary material for: Racial differences in the burden of coronary artery calcium and carotid intima media thickness between Blacks and Whites
Source: Neth Heart J. 2014 Oct 24;23(1):44–51. doi: 10.1007/s12471-014-0610-4 (PMC4268220; doi:10.1007/s12471-014-0610-4)
Supplement: Supplementary file 2 — (DOC 47 kb) [file 12471_2014_610_MOESM2_ESM.doc]

Supplementary table b: Baseline characteristics of 776 participants with available information on coronary artery calcium (CAC) score by sex

| Variable | Male | | Female | | P-value |
| --- | --- | --- | --- | --- | --- |
|  | No of subjects | Mean (SD) or % | No of subjects | Mean (SD) or % |  |
| Total CAC Score | 425 | 214 (489) | 355 | 93 (223) | <0.001 |
| Age (years) | 425 | 60 (7) | 355 | 61 (7) | 0.78 |
| Race – White | 425 | 261 (61%) | 355 | 157 (44%) | <0.001 |
| Race – Black | 425 | 164 (39%) | 355 | 198 (56%) |  |
| Current smoker | 422 | 66 (16%) | 354 | 48 (14%) | 0.66 |
| Diabetes | 423 | 49 (12%) | 353 | 99 (28%) | <0.001 |
| Systolic BP | 425 | 141 (18) | 355 | 146 (21) | <0.001 |
| Body mass index | 420 | 30 (5) | 354 | 32 (6) | <0.001 |
| LDL cholesterol (mg/dl) | 425 | 139 (35) | 355 | 153 (42) | <0.001 |
| HDL cholesterol | 425 | 45 (13) | 355 | 55 (16) | <0.001 |
| Triglycerides (mg/dl) | 425 | 135 (83) | 355 | 150 (104) | 0.02 |
| Fasting glucose | 422 | 102 (28) | 354 | 108 (37) | 0.012 |
| sdLDL (mg/dl) | 425 | 52 (19) | 355 | 50 (22) | 0.17 |
| Log-hsCRP | 392 | 0.17 (1.09) | 337 | 0.77 (1.19) | <0.001 |
| Log-IL-6 | 389 | 0.46 (0.72) | 329 | 0.74 (0.69) | <0.001 |
| CD40L (ng/ml) | 121 | 2.02 (2.41) | 106 | 2.34 (2.36) | 0.33 |
| sICAM-1 (ng/ml) | 348 | 213 (86) | 316 | 232 (107) | 0.009 |
| Endostatin (ng/ml) | 132 | 121 (38) | 113 | 130 (39) | 0.08 |
