# Supplementary material for: Racial differences in the burden of coronary artery calcium and carotid intima media thickness between Blacks and Whites
Source: Neth Heart J. 2014 Oct 24;23(1):44–51. doi: 10.1007/s12471-014-0610-4 (PMC4268220; doi:10.1007/s12471-014-0610-4)
Supplement: Supplementary file 3 — (DOC 35 kb) [file 12471_2014_610_MOESM3_ESM.doc]

Supplementary table 2. The association of traditional cardiovascular risk factors with odds* of significant carotid intima media thickness (> 1mm) and coronary calcification (Agatston Score >100)

| Risk factors | Significant carotid intima media thickness (> 1mm) | | | Significant coronary artery calcification (Agatston Score >100) | | |
| --- | --- | --- | --- | --- | --- | --- |
|  | N | OR (95% CI) | χ^2^ | N | OR (95% CI) | χ^2^ |
| Age (per 1 SD) | 776 | 1.55(1.21,1.98) | 12.3 | 767 | 2.16(1.77,2.65) | 56.0 |
| Sex (Female vs Male) | 776 | 0.39(0.24,0.63) | 14.8 | 767 | 0.31(0.21,0.46) | 32.7 |
| Race (Black vs White) | 776 | 1.70(1.08,2.66) | 5.3 | 767 | 0.49(0.34,0.71) | 14.5 |
| Systolic (per 1 SD) | 776 | 1.31(1.04,1.64) | 5.4 | 767 | 1.25(1.04,1.49) | 5.5 |
| Smoker (Yes vs No) | 776 | 2.06(0.99,4.27) | 3.8 | 767 | 1.31(0.79,2.18) | 1.1 |
| Diabetes (Yes vs No) | 776 | 1.04(0.48,2.25) | 0.0 | 767 | 1.56(0.97,2.49) | 3.4 |
| BMI (per 1 SD) | 776 | 1.18(0.91,1.51) | 1.6 | 767 | 1.24(1.00,1.53) | 3.8 |
| Total cholesterol (per 1 SD) | 776 | 1.24(1.00,1.54) | 3.9 | 767 | 0.98(0.83,1.17) | 0.0 |
| HDL (per 1 SD) | 776 | 0.78(0.61,1.00) | 3.8 | 767 | 0.94(0.76,1.15) | 0.4 |

*Odds ratios for the risk factors shown in the table were mutual adjusted for each other in a multivariable logistic regression model
